# Differences in relative and absolute effectiveness of oral P2Y_12_ inhibition in men and women: a meta-analysis and modelling study

**DOI:** 10.1136/heartjnl-2017-312003

**Published:** 2017-10-05

**Authors:** Kuan Ken Lee, Nicky Welton, Anoop S Shah, Philip D Adamson, Sofia Dias, Atul Anand, David E Newby, Nicholas L Mills, David A McAllister

**Affiliations:** 1 BHF Centre for Cardiovascular Science, University of Edinburgh, Edinburgh, UK; 2 School of Social and Community Medicine, University of Bristol, Bristol, UK; 3 Institute for Health and Wellbeing, University of Glasgow, Glasgow, UK

**Keywords:** acute coronary syndromes, acute myocardial infarction, epidemiology and meta-analysis

## Abstract

**Objective:**

To estimate the absolute treatment effects of newer P2Y_12_ inhibitors (ticagrelor and prasugrel) compared with clopidogrel in men and women with acute coronary syndrome (ACS).

**Methods:**

We searched Ovid MEDLINE, Embase and the Cochrane Central Register of Controlled Trials for randomised controlled trials of oral P2Y_12_ inhibitors for acute stroke or ACS. Age-specific and sex-specific mortality was obtained for all patients admitted to hospital with myocardial infarction in Scotland from 2006 to 2010 (prior to introduction of prasugrel or ticagrelor).

**Results:**

From 9277 articles, nine fulfilled our inclusion criteria. Three trials compared newer P2Y_12_ inhibitors to clopidogrel in ACS, in which the treatment rate ratio (RR) for major adverse cardiovascular events in men was 0.80 (95% CI 0.69 to 0.93). For the same outcome, across all nine trials, the sex–treatment interaction RR was 1.08 (95% CI 0.98 to 1.19). Combining these estimates yielded a treatment RR in women of 0.86 (95% CI 0.72 to 1.04).

17 842 women and 27 818 men were admitted to hospital with myocardial infarction. Mortality was higher for women than men for all-cause (5708, 32.0% vs 5891, 21.2%), cardiovascular (4032, 22.6% vs 4117, 14.8%) and bleeding (193, 1.1% vs 228, 0.8%) deaths.

On applying the sex-specific RRs to this population, the absolute risk reduction for mortality at 1 year was similar for women and men for all-cause (2.30% (95% CI −0.92% to 5.22%) vs 2.47% (95% CI 0.62% to 4.10%)), cardiovascular (2.70% (95% CI −0.63% to 5.74%)) vs 2.72% (95% CI 0.92% to 4.35%)) and bleeding (−0.27% (95% CI −1.06% to 0.30%) vs −0.18% (95% CI −0.71% to 0.24%)) deaths.

**Conclusion:**

Newer P2Y_12_ inhibitors may be slightly less efficacious in women than men, but the absolute risk reduction is similar in both sexes.

## Background

Despite large falls in cardiovascular mortality over the past two decades, overall annual cardiovascular mortality rates remain higher for women than for men.^1^ There are important sex differences in the pathophysiology, clinical presentation and clinical outcomes of women with cardiovascular disease compared with men; women with cardiovascular disease remain an understudied, underdiagnosed and undertreated group.^2–4^

Oral P2Y_12_ inhibitors are a mainstay of treatment for acute coronary syndrome (ACS). They have been evaluated across a broad spectrum of cardiovascular disease in several randomised controlled trials. Consequently, most clinical guidelines recommend P2Y_12_ inhibitors for ACS.^5–8^

A recent individual-level patient data (IPD) meta-analysis compared efficacy of novel P2Y_12_ inhibitors for women and men with stable and acute coronary artery disease, finding very similar relative treatment effects (HRs) for major adverse cardiovascular events, bleeding and all-cause mortality.^9^ Importantly, the study also presented absolute differences in treatment effects in women and men; since women experienced a higher baseline risk of subsequent cardiovascular events, this meant they also received greater overall treatment benefits than men.

However, the absolute treatment effects were also obtained using the trial data, and people with myocardial infarction enrolled in clinical trials are on average younger, less frequently hospitalised, at lower risk of adverse events and less likely to have comorbid disease than people with myocardial infarction identified in disease registers.^10 11^ Moreover, the previous review included patients treated with intravenous P2Y_12_ inhibitors during elective percutaneous coronary intervention as well as patients with stable coronary disease. Consequently, it is not known whether men and women experience similar benefits from P2Y_12_ inhibitors following acute hospitalisations for myocardial infarction.

The objective of this study is therefore to (1) identify whether there exist sex–treatment interactions in the relative efficacy of P2Y_12_ inhibitors and (2) to estimate the effect, in patients typically seen in clinical practice, of any such differences on the absolute treatment benefits obtained from prasugrel or ticagrelor versus clopidogrel.

## Methods

We conducted a systematic review of trials of P2Y_12_ inhibitors in men and women and meta-analysed these data to produce sex-specific estimates of relative treatment efficacy for novel P2Y_12_ inhibitors (prasugrel and ticagrelor) in ACS and acute cerebrovascular disease. We subsequently combined these estimates with data on event rates in a target population that was not exposed to novel P2Y_12_ inhibitors in order to estimate the absolute treatment effect in a real-world population.

Ethical approval was not sought as data were either published or were aggregated at country level by the national organisation responsible for publishing health outcome data.

### Systematic review

#### Search strategy

We searched, without language restriction, using Ovid MEDLINE and Embase (from 1946 to 16 July 2017) and the Cochrane Central Register of Controlled Trials for: ‘ischaemic heart disease’, ‘acute coronary syndrome’, ‘myocardial infarction’, ‘angina’, ‘stroke’, ‘cerebrovascular disease’, ‘P2Y_12_ inhibitor’, ‘clopidogrel’, ‘prasugrel’, ‘ticagrelor’ and ‘randomised controlled trials’ (detailed search strategy in online supplementary appendix). We also hand-searched the bibliography of included studies and relevant review articles.

10.1136/heartjnl-2017-312003.supp1 Supplementary appendix

#### Selection of articles and extraction of data

Studies were included if they met the following inclusion criteria: (1) randomised controlled trials of oral P2Y_12_ inhibitors in ACS or stroke and (2) trials providing data on major adverse cardiovascular events stratified by sex. We excluded randomised controlled trials (RCTs) of intravenous P2Y_12_ inhibitors and RCTs that evaluated different durations of P2Y_12_ therapy. One investigator (KKL) performed initial screening of titles and abstracts. A random sample of 200 titles and abstracts were assessed during the initial screening process by a second investigator (AS) without disagreements. Two investigators examined the full-text reports for eligibility according to our prespecified review protocol. Data extraction was carried out independently by KKL and AS and adjudicated by AA.

#### Definition of outcomes

The clinical outcome examined in this review was the composite endpoint ‘major adverse cardiovascular events’ (MACE) reported as the primary efficacy endpoint in each individual trial (table 1). Outcomes include elements of cardiovascular mortality, all-cause mortality, myocardial infarction, stroke (ischaemic or haemorrhagic) and occluded infarct-related coronary artery. We also examined the safety outcome of bleeding as defined by the individual studies that used either standardised Thrombolysis in Myocardial Infarction (TIMI) or Global Utilization of Streptokinase and Tissue Plasminogen Activator for Occluded Coronary Arteries definitions, or trial specific criteria (table 1).

**Table 1:** Design of trials included in meta-analysis

Trial, year	n	Population	Drug/comparator	Dosage	Duration of treatment	Primary efficacy endpoint	Primary safety endpoint	Follow-up
CURE, 2001	12 562	Patients presenting to hospital with acute coronary syndrome* within 24 hours after onset of symptoms and did not have ST elevation	Clopidogrel/placebo	300 mg loading dose followed by 75 mg once daily	3–12 months (mean duration of treatment, 9 months)	CV death, non-fatal MI or stroke	Major bleeding (requiring transfusion of ≥2 units of blood)	12 months
COMMIT, 2005	45 852	Patients admitted within 24 hours of suspected acute MI onset with ST elevation, left-bundle branch block or ST depression.	Clopidogrel/placebo	75 mg	Hospital discharge or 28 days (mean 14.9 days)	Death, MI or stroke	Haemorrhagic stroke or non-cerebral bleeding (requiring transfusion or fatal)	Hospital discharge or 28 days.
CLARITY-TIMI 28, 2005	3491	Patients 18–75 years of age presenting within 12 hours after onset of STEMI	Clopidogrel/placebo	300 mg loading dose followed by 75 mg once daily	Up to day of coronary angiography, day 8 or hospital discharge	Death, MI or occluded infarct-related artery (TIMI flow grade of 0 or 1).	TIMI major bleeding	30 days
TRITON-TIMI 38, 2007	13 608	Patients with acute coronary syndrome* with scheduled PCI	Prasugrel/clopidogrel	60 mg loading dose followed by 10 mg once daily	6–15 months (median, 14.5 months)	CV death, non-fatal MI or non-fatal stroke	TIMI non-CABG major bleeding	15 months
PLATO, 2009	18 624	Patients hospitalised for an acute coronary syndrome* with an onset of symptoms during previous 24 hours	Ticagrelor/clopidogrel	180 mg loading dose followed by 90 mg twice daily	12 months	CV death, MI or stroke	Study defined major bleeding	12 months
CHANCE, 2010	5170	Patients within 24 hours after the onset of minor ischaemic stroke or high-risk TIA	Clopidogrel/placebo	300 mg loading dose followed by 75 mg once daily	90 days	New stroke (ischaemic or haemorrhagic)	GUSTO moderate to severe bleeding	90 days
TRILOGY ACS, 2012	7243	Patients with unstable angina* or NSTEMI selected for medical management without revascularisation within 10 days after the index event	Prasugrel/clopidogrel	30 mg loading dose followed by 10 mg once daily	30 months	CV death, non-fatal MI or non-fatal stroke	TIMI non-CABG major bleeding	30 months
SPS3, 2012	3020	Patients with recent symptomatic lacunar infarcts identified by MRI.	Clopidogrel/placebo	75 mg	3.4 years	Stroke recurrence (ischaemic stroke or intracranial haemorrhage, including subdural haematomas)	Study defined major extracranial haemorrhage	3.4 years
SOCRATES, 2016	13 199	Patients with a non-severe ischaemic stroke or high-risk transient ischaemic attack who had not received intravenous or intra-arterial thrombolysis and were not considered to have had a cardioembolic stroke	Ticagrelor/aspirin	180 mg loading dose followed by 90 mg twice daily	90 days	Stroke, MI or death	PLATO major bleeding	90 days

*For trials that included patients with unstable angina as well as MI (CURE, PLATO, TRITON-TIMI 38 and TRILOGY ACS), the overall treatment effect estimate and the effect estimates having excluded patients with unstable angina were very similar (online supplementary appendix for additional detail).

Trial acronyms: CHANCE, Clopidogrel in High-Risk Patients with Acute Non-disabling Cerebrovascular Events Trial; CLARITY-TIMI 28, Clopidogrel as Adjunctive Reperfusion Therapy-Thrombolysis in Myocardial Infarction 28 Study; COMMIT, Clopidogrel and Metoprolol in Myocardial Infarction Trial; CURE, Clopidogrel in Unstable Angina to Prevent Recurrent Events Trial; PLATO, Platelet Inhibition and Patient Outcomes Trial; SPS3, Secondary Prevention of Small Subcortical Strokes Trial; SOCRATES, Acute Stroke or Transient Ischaemic Attack Treated with Aspirin or Ticagrelor and Patient Outcomes; TRILOGY ACS, Targeted Platelet Inhibition to Clarify the Optimal Strategy to Medically Manage Acute Coronary Syndromes; TRITON-TIMI 38, Trial to Assess Improvement in Therapeutic Outcomes by Optimising Platelet Inhibition with Prasugrel-Thrombolysis in Myocardial Infarction 38.

CABG, coronary artery bypass grafting; CV, cardiovascular; GUSTO, Global Utilization of Streptokinase and Tissue Plasminogen Activator for Occluded Coronary Arteries; MI, myocardial infarction; NSTEMI, non-ST elevation myocardial infarction; PCI, percutaneous coronary intervention; STEMI, ST elevation myocardial infarction; TIA, transient ischaemic attack; TIMI, Thrombolysis in Myocardial Infarction.

## Target population

NHS National Services Scotland provided data on deaths at 1 year following a hospital admission with myocardial infarction from the national linked hospitalisation and death database. Myocardial infarction admissions were those assigned a WHO 10th International Classification of Diseases 10 code (ICD-10) of I21 or I22.^12^ All people in Scotland aged 30–99 years who had such an admission during the 5-year time period from 2006 to 2010 were included. This period was chosen as neither prasugrel nor ticagrelor was available at that time, such that aspirin and clopidogrel was the standard treatment for non-ST elevation myocardial infarction. Where patients had multiple admissions during this period, only the first episode was included.

Deaths were defined as cardiovascular (I00–I99), bleeding (see online supplementary appendix) or non-bleeding, non-cardiovascular deaths. Bleeding codes were selected based on those used to identify Bleeding Academic Research Consortium^13^ bleeding by the Clinical research using LInked Bespoke studies and Electronic health Records group for use in the MINAP database.^14^ Deaths for which both bleeding and cardiovascular codes had been recorded were defined as bleeding deaths for the purpose of this analyses.

## Statistical analysis

Trials were categorised by indication (ACS or stroke) with ACS trials being further split by treatment (newer P2Y_12_ inhibitors vs clopidogrel or clopidogrel vs placebo). We extracted number of events and total number randomised for each treatment broken down by sex for each trial, fitting a hierarchical generalised linear model with a binomial likelihood and a cumulative log–log link. The model estimates relative treatment effects, directly comparing greater versus weaker/absent P2Y_12_ inhibition (ticagrelor/prasugrel>clopidogrel>placebo), with terms for the intercept, sex and treatment, as well as a sex–treatment interaction term to allow for sex-specific treatment effects. We explored three different models for the interaction: an identical interaction for all trials within the drug class, shared interactions between trials within the drug class (using the assumption of exchangeability of covariate–treatment interactions within drug class^15^) and shared interactions between trials having stratified by indication/treatment. We used the posterior mean deviance and deviance information criteria (DIC) to choose between models, preferring models with lower values of dbar and DIC.

The analysis was run separately for MACE and bleeding outcomes. For the MACE outcome, minimally informative priors were used. Due to small amounts of data for the bleeding outcome, we specified an informative prior to constrain the magnitude of the estimated sex–treatment interaction (normal distribution, mean=0, SD=0.114), and only ran the shared interaction model.

**Figure 1:**
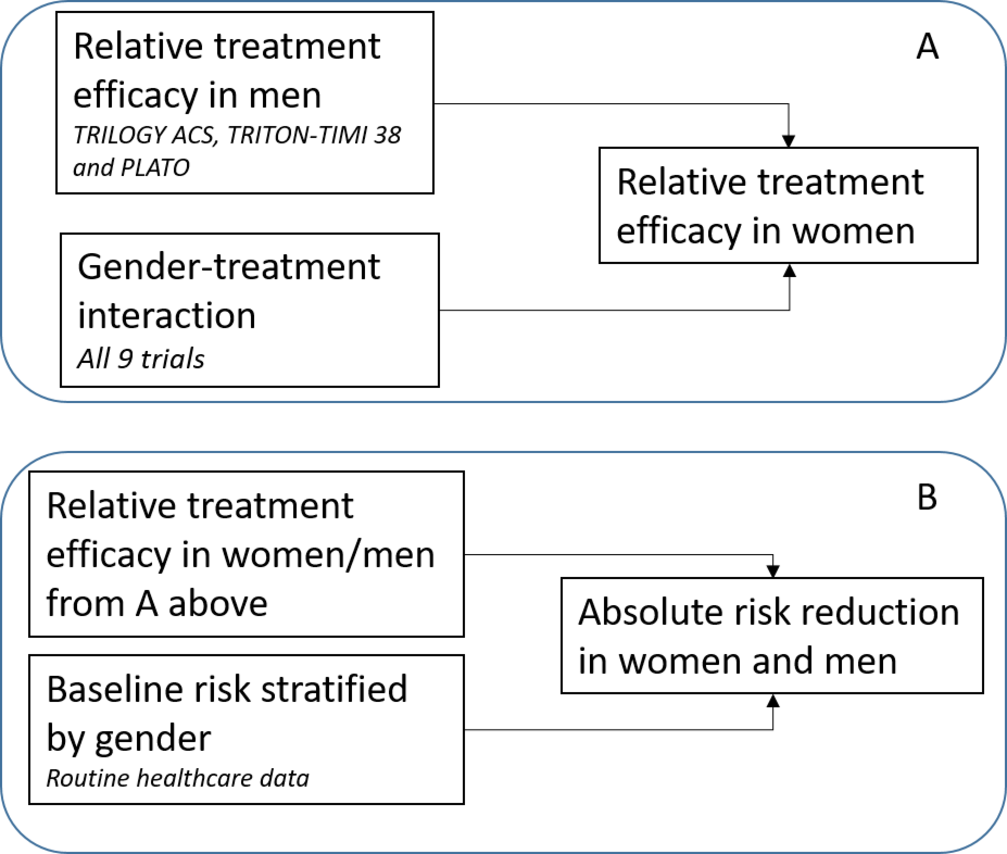
Overview of data sources used in modelling. Panel A shows the data sources used to estimate the relative treatment efficacy of newer P2Y_12_ inhibitors in women and men and panel B shows the data sources used to estimate the absolute treatment effect. PLATO, Platelet Inhibition and Patient Outcomes Trial; TRILOGY ACS, Targeted Platelet Inhibition to Clarify the Optimal Strategy to Medically Manage Acute Coronary Syndromes; TRITON-TIMI 38, Trial to Assess Improvement in Therapeutic Outcomes by Optimising Platelet Inhibition with Prasugrel-Thrombolysis in Myocardial Infarction 38.

For both MACE and bleeding, sex-specific relative treatment effect estimates for prasugrel or ticagrelor versus clopidogrel were obtained by combining the overall sex–treatment interaction derived from all included trials with the treatment effect in men for trials of prasugrel or ticagrelor versus clopidogrel (figure 1A).

The absolute risk reduction (ARR) for women and men was obtained by combining these sex-specific relative treatment efficacy estimates with baseline risk data for the Scottish population (figure 1B). We estimated the baseline rates according to age and sex for bleeding deaths, cardiovascular deaths and non-bleeding, non-cardiovascular deaths from the Scottish data using a generalised linear model with a log-link and multinomial distribution to simultaneously estimate the multiple outcomes accounting for competing risks. The treatment efficacy rate ratios (RRs) from the synthesis of the trial data were used to modify the parameters of the baseline risk model to obtain ARR estimates. In extensive sensitivity analyses, we varied the baseline rates and sex–treatment interactions for MACE and bleeding.

All analyses were carried out within a Bayesian framework, which meant that combining the estimates from the trial and observational data was straightforward.^16^ Model chains were examined graphically to investigate mixing and convergence. All analyses were conducted in R (V.3.3.1) and JAGS (V.4.2.0). All data and analysis codes are available at (https://github.com/dmcalli2/gender_dapt_manuscript) with selected code and model descriptions provided in the online supplementary material.

## Results

### Systematic review

We assessed the abstracts of 9277 articles identified through database searching (online supplement). Forty-one potentially relevant articles were reviewed in detail. Of these, we identified nine articles that fulfilled our inclusion criteria.^17–25^

All nine studies were double-blind, randomised-controlled trials. CURE, COMMIT and CLARITY-TIMI 28 compared clopidogrel with placebo in ACS, CHANCE and SPS3 compared clopidogrel with placebo in stroke, SOCRATES compared ticagrelor with aspirin in stroke and TRITON-TIMI 38, PLATO and TRILOGY ACS compared the novel P2Y_12_ antagonists with clopidogrel in ACS. The duration of follow-up ranged from 2 weeks (COMMIT) to 3 years (SPS3) (table 1).

The average age of patients enrolled was similar across all trials (online supplementary appendix). However, women were under-represented. In the Scottish population, 39.0% (17 842/45 660) of people admitted with myocardial infarction were women, whereas only 29.7% (32 561/109 570) of participants enrolled overall were women (for each trial, the proportion of women was 19.7%, 26.0%, 27.8%, 28.4%, 33.8%, 35.9%, and 37.0%, respectively).

### Meta-analysis

#### Sex–treatment interaction

**Figure 2:**
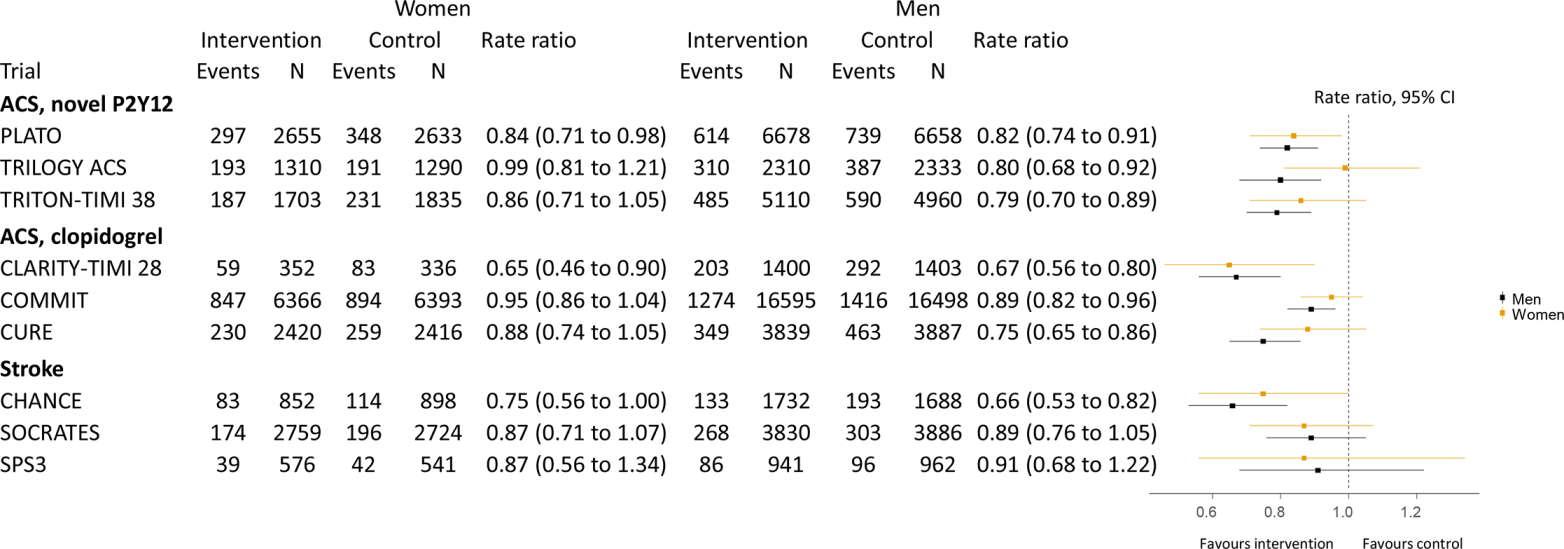
Relative treatment efficacy for cardiovascular events stratified by sex. Rate ratios and 95% credible intervals estimates of sex-specific treatment effects for each trial. Estimates for each trial were estimated independently in generalised linear models. Points represent the point estimates, and bars represent the 95% credible intervals. ACS, acute coronary syndrome.

On comparing the three models of sex–treatment interactions across all nine trials of P2Y_12_ inhibitors (figure 2), the model assuming a shared sex–treatment interaction for the whole drug class had both a lower deviance and DIC than the other models (the deviance was 273, 261 and 259 and the DIC was 291, 289 and 286 for the identical, stratified and shared models, respectively), indicating better fit. Consequently, this model was preferred.

**Figure 3:**
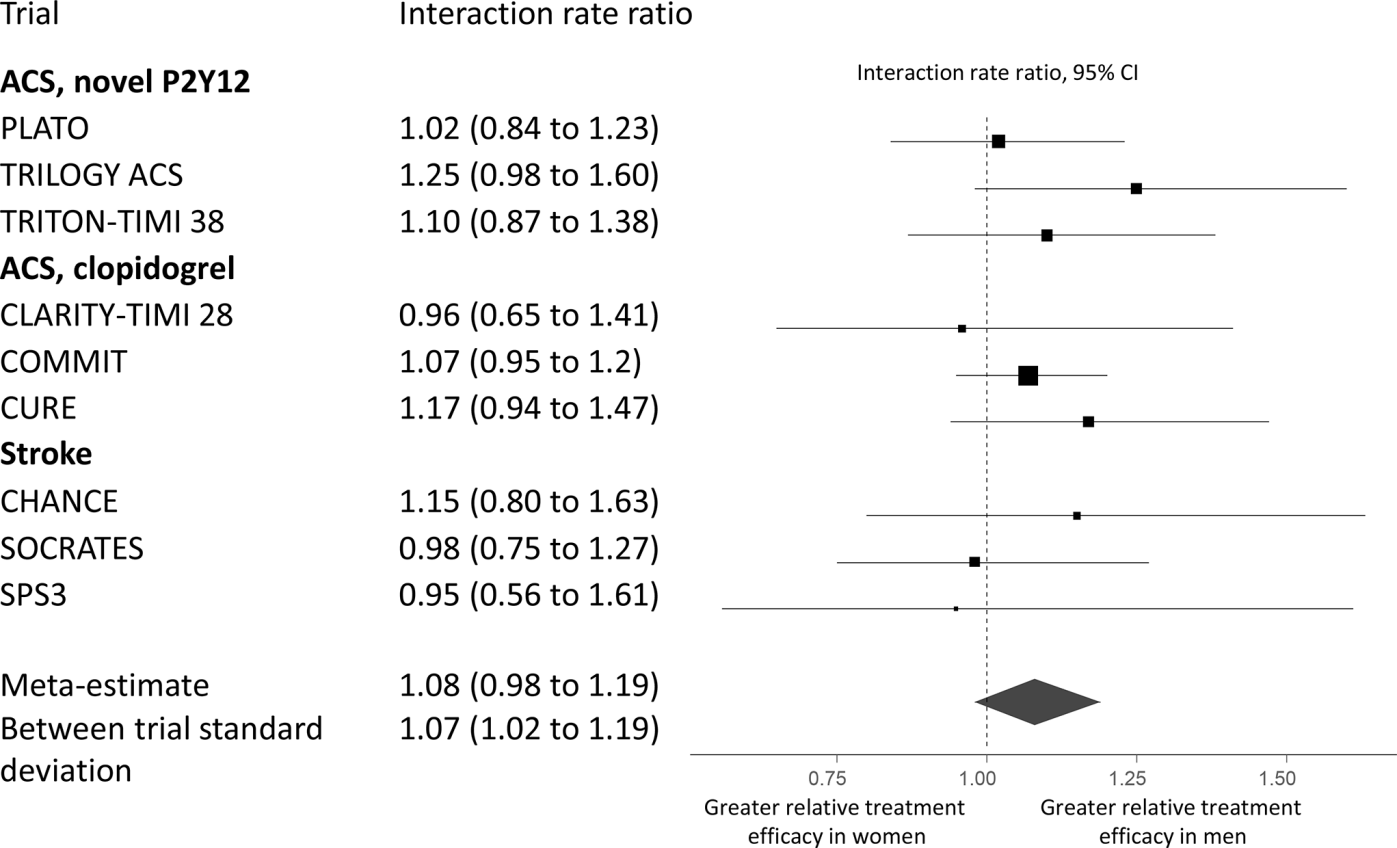
Relative treatment efficacy for cardiovascular events. Meta-estimates and 95% credible intervals estimates of sex–treatment interactions for each trial as well as the overall sex–treatment interaction for P2Y_12_ inhibitors. All estimates were obtained from generalised linear models. The overall sex–treatment interaction was estimated in a hierarchical model. Points represent the point estimates, and bars represent the 95% credible intervals. The point-size for each trial is proportional to the inverse of the SE squared for each interaction estimate. The vertices of the diamond indicate the 95% credible intervals for the meta-estimate, which was obtained from the ‘shared’ model. ACS, acute coronary syndrome.

For this shared-effects model, the RR for the sex–treatment interaction was 1.08 (95% credible interval (CI) 0.98 to 1.19, figure 3). Similar estimates for the sex–treatment interactions were found for the identical (RR 1.09; 95% CI 1.01 to 1.17) and stratified (RR for prasugrel/ticagrelor vs clopidogrel comparison 1.11; 95% CI 0.93 to 1.32) models.

The treatment effect RR in men for the three trials comparing prasugrel or ticagrelor to clopidogrel in ACS was 0.80 (95% CI 0.69 to 0.93). Combining this estimate with the sex–treatment interaction estimate from the shared model yielded an RR in women of 0.86 (95% CI 0.72 to 1.04). This meant the relative risk reduction was approximately 30% lower in women than in men.

For bleeding, the sex–treatment interaction RR was 1.04 (95% CI 0.88 to 1.21). This estimate did not change importantly from the moderately informative prior we used in the modelling for this outcome (prior for sex–treatment interaction on RR scale 1.00; 95% CI 0.80 to 1.25). In men, the treatment effect on bleeding was RR 1.13 (95% CI 0.75 to 1.98). As such, the modelled treatment effect on bleeding in women was RR 1.18 (95% CI 0.76 to 2.08).

#### Sex-specific absolute treatment effects in the Scottish population

**Figure 4:**
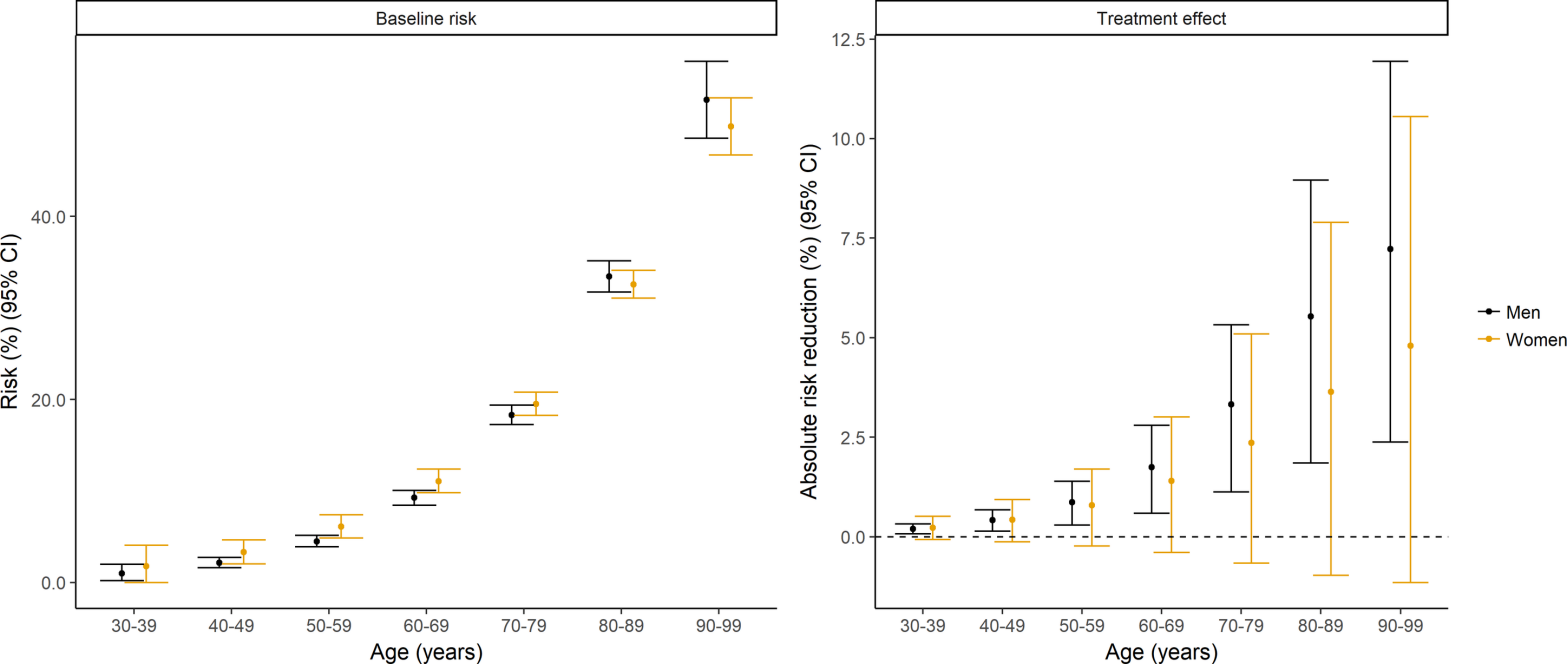
Baseline risk of death and estimates of the absolute treatment effects of oral P2Y_12_ inhibition in women and men with myocardial infarction in Scotland. Panel A shows estimated risks of deaths obtained from a regression model run on the national Scottish data. A generalised linear model with a log-link and multinomial distribution was fitted to all three outcomes (cardiovascular death, bleeding death and non-cardiovascular, non-bleeding death), which included parameters for age and sex as well as age–sex interactions. Panel B shows the modelled absolute risk reduction in this population, obtained from applying to this model the relative treatment efficacy for cardiovascular events and bleeding events in men and women. Most patients in the clinical trial data were aged less than 75 years, and we were unable to examine whether treatment effects showed heterogeneity by age. As such, estimates for older patients should be treated with caution.

A total of 17 842 women and 27 818 men were admitted to hospital with myocardial infarction. Mortality was higher for women than men for all-cause mortality (5708, 32.0% vs 5891, 21.2%) and cardiovascular mortality (4032, 22.6% vs 4117, 14.8%). Mortality related to bleeding was also commoner in women (193, 1.1% vs 228, 0.8%) deaths. The sex differences in cardiovascular mortality differed by age with younger women having higher mortality than men and older women having lower mortality than men (table 2, figure 4).

**Table 2:** Scotland, mortality at 1 year following hospitalisation for myocardial infarction from 2006 to 2010

Age (years)	Sex	Population	All cause	Cardiovascular	Bleeding	Other*
30–39	Female	147	12 (8.2)	8 (5.4)	0 (0.0)	4 (2.7)
	Male	507	10 (2.0)	8 (1.6)	0 (0.0)	2 (0.4)
40–49	Female	793	38 (4.8)	22 (2.8)	0 (0.0)	16 (2.0)
	Male	2928	105 (3.6)	77 (2.6)	6 (0.2)	22 (0.8)
50–59	Female	1681	137 (8.1)	84 (5.0)	13 (0.8)	40 (2.4)
	Male	5513	315 (5.7)	228 (4.1)	18 (0.3)	69 (1.3)
60–69	Female	3109	486 (15.6)	312 (10.0)	13 (0.4)	161 (5.2)
	Male	6688	815 (12.2)	563 (8.4)	32 (0.5)	220 (3.3)
70–79	Female	5066	1475 (29.1)	990 (19.5)	48 (0.9)	437 (8.6)
	Male	6837	1923 (28.1)	1299 (19.0)	67 (1.0)	557 (8.1)
80–89	Female	5518	2621 (47.5)	1933 (35.0)	91 (1.6)	597 (10.8)
	Male	4653	2256 (48.5)	1600 (34.4)	90 (1.9)	566 (12.2)
90–99	Female	1528	939 (61.5)	683 (44.7)	28 (1.8)	228 (14.9)
	Male	692	467 (67.5)	342 (49.4)	15 (2.2)	110 (15.9)
All ages	Female	17 842	5708 (32.0)	4032 (22.6)	193 (1.1)	1483 (8.3)
	Male	27 818	5891 (21.2)	4117 (14.8)	228 (0.8)	1546 (5.6)
Both		45 660	11 599 (25.4)	8149 (17.8)	421 (0.9)	3029 (6.6)

n (%) deaths.

*Non-bleeding, non-cardiovascular.

Based on our model, the ARR in all-cause mortality at 1 year was similar in both sexes. For the 27 818 men admitted to hospital with myocardial infarction, the modelled ARR was 2.72% (95% CI 0.92 to 4.35) for cardiovascular, −0.18% (95% CI −0.71 to 0.24) for bleeding and 2.47% (95% CI 0.62 to 4.10) for all-cause mortality. For the 17 842 women, the ARRs were similar, 2.70% (95% CI −0.63 to 5.74) for cardiovascular, −0.27% (95% CI −1.06 to 0.30) for bleeding and 2.30% (95% CI −0.92 to 5.22) for all-cause mortality. In contrast with the 0.93 probability that relative treatment efficacy is lower in women than men, the probability that the ARR for all-cause mortality was lower in women than men was only 0.53.

In a subsequent analysis, we modelled the absolute treatment effect assuming that the relative treatment effect was the same in both sexes. Under this alternative model, a larger benefit was seen in women (3.23%; 95% CI 0.84 to 5.41) than in men (2.21%; 95% CI 0.56 to 3.72).

#### Age-specific and sex-specific absolute treatment effects

The same pattern was evident when the absolute treatment effects were modelled within each age group (figure 4). Assuming no age–treatment interaction, much larger ARRs were seen in older people than in younger people. For example, the ARR was 0.20% (95% CI 0.07 to 0.32) and 3.32% (95% CI 1.12 to 5.32) in men aged 30–39 and 70–79 years, respectively.

#### Sensitivity analyses

We modelled the absolute treatment effect for a range of different event rates for bleeding deaths in women and men, allowing for bleeding rates up to 10-fold higher in men than observed and up to twofold higher in women than in men. The point estimate for the overall absolute treatment effect in women remained consistently around 2% (range 1.92% to 2.16%).

## Discussion

In a meta-analysis and modelling study using data from 100 000 participants randomised to P2Y_12_ inhibitors and over 45 000 patients from the Scottish population hospitalised with myocardial infarction, we examined relative and absolute treatment effects for newer P2Y_12_ inhibitors.

For relative treatment effects, the results were equivocal, although more supportive of P2Y_12_ inhibitors being less effective in women than men. Importantly, however, the baseline risk of subsequent cardiovascular death following myocardial infarction was higher in women. Therefore, in absolute terms, women and men had a similar benefit from treatment. Indeed, while there was a 93% probability that the relative treatment effect was lower in women, the probability for the absolute treatment effect being lower was only 53%. This suggests that women are likely to experience similar benefits to men when treated with newer P2Y_12_ inhibitors.

There has been considerable interest in differences in treatment effects from P2Y_12_ inhibitors in women and men, which could arise from a range of causes. The spectrum of disease may be different; women with myocardial infarction present later than men and with different clinical and diagnostic features.^4^ Underlying differences in cardiovascular biology have also been implicated.^26^ Women have smaller coronary arteries and different carotid anatomy compared with their male counterparts.^27^ Women are also less likely to display thrombogenic tendencies, and platelet–subendothelial interactions are markedly reduced following antiplatelet therapy in men compared with women.^28^ Some of these differences are likely to be related to the hormonal milieu.^26^

Nonetheless, previous studies examining variation in relative treatment effects for antiplatelet agents have yielded either null or small sex–treatment interactions. A previous IPD meta-analysis of five studies examining the effect of clopidogrel in the secondary prevention of cardiovascular disease (including three of the studies in our meta-analysis) found that clopidogrel was around 1.1-fold less efficacious in women for both cardiovascular events and mortality.^29^ A more recent IPD meta-analysis examining the efficacy of ticagrelor, prasugrel or cangrelor in men and women with coronary artery disease (three of the six trials were included in our meta-analysis) found no statistically significant difference in major adverse cardiovascular events.^9^ The report did not include the estimate and CIs for the sex–treatment interaction. However, we calculated these using the published data (see online supplementary material), finding a relative difference in HR of 1.00 (95% CI 0.86 to 1.14),^9^ a range which includes our own estimate of 1.08.

One strength of our study over previous meta-analyses is that we included additional trials from within the drug class (ie, stroke trials and trials comparing clopidogrel with placebo), allowing us to more precisely estimate the sex–treatment interactions while still allowing for differences by indication and treatment in the modelling. However, this approach is unlikely to account for the different findings as on restricting the efficacy estimates to trials of ticagrelor versus clopidogrel for ACS; we also found a 1.1-fold difference between men and women. Consequently, the most plausible reason for the variation across meta-analyses is the difference in included trials.

Nonetheless, in neither our study nor any meta-analysis of randomised controlled trials of which we are aware was there a difference large enough to outweigh the greater baseline risk in women. In the Scottish population, a sex–treatment interaction of around 1.2-fold lower efficacy in women would be needed for women to have a substantially lower ARR than men. Since the treatment effect in men is around 0.80, this amounts to a qualitative interaction, that is, an interaction that changes the direction of effect not just the magnitude. The risk of cardiovascular mortality following myocardial infarction has been found to be higher in woman than men in other settings,^1 2 30^ to which this finding is likely to be applicable.

The assumptions we employed in this modelling study are provided in detail in the online supplementary appendix. We discuss three important assumptions here. First, we assumed that the included trials were sufficiently similar so as to be exchangeable in terms of sex–treatment interactions. This assumption is, we would argue, considerably weaker than the assumption that the main effect is consistent across trials. Second, some patients admitted to hospital in Scotland with myocardial infarction during the 5-year period will not have received dual antiplatelet therapy. Nonetheless, in an all-comers trial of diagnostic practice in patients with ACS in 2012 in a large region of Scotland, we found that 76% of patients received dual antiplatelet therapy.^31^ More importantly, as we did not have access to IPD, we could not examine heterogeneity in treatment effect by age or by age and sex in combination. As such, considerable caution is needed in applying these results to older people.

## Conclusion

In a meta-analysis and modelling study using data from 100 000 participants randomised to P2Y_12_ inhibitors and over 45 000 patients from the Scottish population hospitalised with myocardial infarction, we have identified that newer P2Y_12_ inhibitors may be slightly less efficacious in women than men but that the ARR is similar in both sexes.

**Key messages**

**What is already known on this subject?**

Women with myocardial infarction present later than men and with different clinical and diagnostic features. Meta-analyses comparing the effect of clopidogrel and novel P2Y_12_ inhibitors have not estimated the absolute treatment effects for women and men in typical real-world populations.

**What might this study add?**

This study uses a combination of clinical trial and routine healthcare data to show that while P2Y_12_ inhibitors may be slightly less efficacious in women than men, the absolute treatment benefit (in terms of the absolute risk reduction) is similar in both sexes.

**How might this impact on clinical practice?**

This study provides reassurance that mere quantitative sex–treatment interactions (ie, where the magnitude only and not the direction of the effect differs between men and women) are unlikely to result in women experiencing less benefits from secondary prevention than men following myocardial infarction.

## References

[1] Mozaffarian D, Benjamin EJ, Go AS, et al Heart disease and stroke statistics--2015 update: a report from the American Heart Association. Circulation 2015;131:e29–322. 10.1161/CIR.0000000000000152 25520374

[2] Vaccarino V, Parsons L, Every NR, et al Sex-based differences in early mortality after myocardial infarction. N Engl J Med Overseas Ed 1999;341:217–25. 10.1056/NEJM199907223410401 10413733

[3] Wenger NK Women and coronary heart disease: a century after herrick. Circulation 2012;126:604–11. 10.1161/CIRCULATIONAHA.111.086892 22850362

[4] Mehta LS, Beckie TM, DeVon HA, et al Acute myocardial infarction in women: a scientific statement from the American Heart Association. Circulation 2016;133:CIR–0000000000000351 10.1161/CIR.0000000000000351 26811316

[5] Steg PG, James SK, Atar D, et al ESC Guidelines for the management of acute myocardial infarction in patients presenting with ST-segment elevation. Eur Heart J 2012;33:2569–619. 10.1093/eurheartj/ehs215 22922416

[6] Roffi M, Patrono C, Collet JP, et al 2015 ESC Guidelines for the management of acute coronary syndromes in patients presenting without persistent ST-segment elevation: task force for the management of acute coronary syndromes in patients presenting without persistent ST-segment elevation of the European Society of Cardiology (ESC). Eur Heart J 2016;37:267–315. 10.1093/eurheartj/ehv320 26320110

[7] O’Gara PT, Kushner FG, Ascheim DD, et al ACCF/AHA guideline for the management of ST-elevation myocardial infarction. Circulation 2012:CIR.0b013e3182742cf6.

[8] Amsterdam EA, Wenger NK, Brindis RG, et al 2014 AHA/ACC guideline for the management of patients with non–ST-elevation acute coronary syndromes. Circulation 2014;130:e344–426. 10.1161/CIR.0000000000000134 25249585

[9] Lau ES, Braunwald E, Murphy SA, et al Potent P2Y12 inhibitors in men versus women: a collaborative meta-analysis of randomized trials. J Am Coll Cardiol 2017;69:1549–59. 10.1016/j.jacc.2017.01.028 28335837

[10] Udell JA, Wang TY, Li S, et al Clinical trial participation after myocardial infarction in a national cardiovascular data registry. JAMA 2014;312:841–3. 10.1001/jama.2014.6217 25157729

[11] Coca SG, Krumholz HM, Garg AX, et al Underrepresentation of renal disease in randomized controlled trials of cardiovascular disease. JAMA 2006;296:1377–84. 10.1001/jama.296.11.1377 16985230

[12] Anon. Computerised record linkage: compared with traditional patient follow-up methods in clinical trials and illustrated in a prospective epidemiological study. The West of Scotland Coronary Prevention Study Group. J Clin Epidemiol 1995;48:1441–52. 8543958 10.1016/0895-4356(95)00530-7

[13] Mehran R, Rao SV, Bhatt DL, et al Standardized bleeding definitions for cardiovascular clinical trials: a consensus report from the Bleeding Academic Research Consortium. Circulation 2011;123:2736–47. 10.1161/CIRCULATIONAHA.110.009449 21670242

[14] Denaxas SC, George J, Herrett E, et al Data resource profile: cardiovascular disease research using linked bespoke studies and electronic health records (CALIBER). Int J Epidemiol 2012;41:1625–38. 10.1093/ije/dys188 23220717 PMC3535749

[15] Phillippo DM, Ades AE, Dias S, et al Nice dsu technical support document 18: methods for population-adjusted indirect comparisons in submissions to nice. 2016 http://scharr.dept.shef.ac.uk/nicedsu/wp-content/uploads/sites/7/2017/05/Population-adjustment-TSD-FINAL.pdf (accessed 27 Apr 2017).

[16] Dias S, Sutton AJ, Welton NJ, et al NICE DSU Technical Support Document 6:Embedding Evidence Synthesis in Probabalistic Cost-Effectiveness Analysis: Software Choices. 2011 http://scharr.dept.shef.ac.uk/nicedsu/wp-content/uploads/sites/7/2016/03/TSD6-Software.final_.08.05.12.pdf (accessed 24 Jan 2014). 27905718

[17] Yusuf S, Zhao F, Mehta SR, et al Effects of clopidogrel in addition to aspirin in patients with acute coronary syndromes without ST-segment elevation. N Engl J Med 2001;345:494–502. 10.1056/NEJMoa010746 11519503

[18] Chen ZM, Jiang LX, Chen YP, et al Addition of clopidogrel to aspirin in 45,852 patients with acute myocardial infarction: randomised placebo-controlled trial. Lancet 2005;366:1607–21. 10.1016/S0140-6736(05)67660-X 16271642

[19] Sabatine MS, Cannon CP, Gibson CM, et al Addition of clopidogrel to aspirin and fibrinolytic therapy for myocardial infarction with ST-segment elevation. N Engl J Med 2005;352:1179–89. 10.1056/NEJMoa050522 15758000

[20] Wiviott SD, Braunwald E, McCabe CH, et al Prasugrel versus clopidogrel in patients with acute coronary syndromes. N Engl J Med 2007;357:2001–15. 10.1056/NEJMoa0706482 17982182

[21] Wallentin L, Becker RC, Budaj A, et al Ticagrelor versus clopidogrel in patients with acute coronary syndromes. N Engl J Med 2009;361:1045–57. 10.1056/NEJMoa0904327 19717846

[22] Benavente OR, Hart RG, McClure LA, Investigators TS, et al Effects of clopidogrel added to aspirin in patients with recent lacunar stroke. N Engl J Med 2012;367:817–25. 10.1056/NEJMoa1204133 22931315 PMC4067036

[23] Wang Y, Wang Y, Zhao X, et al Clopidogrel with aspirin in acute minor stroke or transient ischemic attack. N Engl J Med 2013;369:11–19. 10.1056/NEJMoa1215340 23803136

[24] Johnston SC, Amarenco P, Albers GW, et al Ticagrelor versus aspirin in acute stroke or transient ischemic attack. N Engl J Med 2016;375:35–43. 10.1056/NEJMoa1603060 27160892

[25] Roe MT, Armstrong PW, Fox KA, Kaa F, et al Prasugrel versus clopidogrel for acute coronary syndromes without revascularization. N Engl J Med 2012;367:1297–309. 10.1056/NEJMoa1205512 22920930

[26] Levin RI The puzzle of aspirin and sex. N Engl J Med 2005;352:1366–8. 10.1056/NEJMe058051 15755763

[27] Herity NA, Lo S, Lee DP, et al Effect of a change in gender on coronary arterial size: a longitudinal intravascular ultrasound study in transplanted hearts. J Am Coll Cardiol 2003;41:1539–46. 12742295 10.1016/s0735-1097(03)00246-8

[28] Escolar G, Bastida E, Garrido M, et al Sex-related differences in the effects of aspirin on the interaction of platelets with subendothelium. Thromb Res 1986;44:837–47. 10.1016/0049-3848(86)90029-0 3798423

[29] Berger JS, Bhatt DL, Cannon CP, et al The relative efficacy and safety of clopidogrel in women and men a sex-specific collaborative meta-analysis. J Am Coll Cardiol 2009;54:1935–45. 10.1016/j.jacc.2009.05.074 19909874

[30] Vaccarino V, Krumholz HM, Yarzebski J, et al Sex differences in 2-year mortality after hospital discharge for myocardial infarction. Ann Intern Med 2001;134:173–81. 10.7326/0003-4819-134-3-200102060-00007 11177329

[31] Shah AS, Griffiths M, Lee KK, et al High sensitivity cardiac troponin and the under-diagnosis of myocardial infarction in women: prospective cohort study. BMJ 2015;350:g7873 10.1136/bmj.g7873 25609052 PMC4301191

