# Supplementary material for: Differences in relative and absolute effectiveness of oral P2Y12 inhibition in men and women: a meta-analysis and modelling study
Source: Heart. 2017 Oct 5;104(8):657–64. doi: 10.1136/heartjnl-2017-312003 (PMC5890639; doi:10.1136/heartjnl-2017-312003)
Supplement: Supplementary appendix [file heartjnl-2017-312003supp001.pdf]

Supplementary appendix

15 August 2017

Search strategy

Medline search strategy

(1946 to July 2017)

ID	Search
1	exp Thienopyridines/
2	exp Purinergic P2Y Receptor Antagonists/
3	(clopidogrel or prasugrel or ticagrelor).tw,nm.
4	(ADP adj3 receptor antagonist*).tw,ot.
5	1 or 2 or 3 or 4
6	Randomized Controlled Trials as Topic/
7	randomized controlled trial/
8	Random Allocation/
9	Double Blind Method/
10	Single Blind Method/
11	clinical trial/
12	controlled clinical trial.pt.
13	randomized controlled trial.pt.
14	clinical trial.pt.
15	exp Clinical Trials as topic/
16	(clinical adj trial$).tw.
17	((singl$ or doubl$ or treb$ or tripl$) adj (blind$3 or mask$3)).tw.
18	PLACEBOS/
19	placebo$.tw.
20	randomly allocated.tw.
21	(allocated adj2 random$).tw.
22	case report.tw.
23	letter/
24	historical article/
25	exp animal/ not humans/
26	review/
27	6 or 7 or 8 or 9 or 10 or 11 or 12 or 13 or 14 or 15 or 16 or 17 or 18 or 19 or 20 or 21
28	22 or 23 or 24 or 25 or 26
29	27 not 28
30	((cerebrovascular disorders/ or basal ganglia cerebrovascular disease/ or brain ischemia/ or exp brain infarction/ or hypoxia-ischemia, brain/ or carotid artery diseases/ or carotid artery thrombosis/ or carotid artery, internal, dissection/ or intracranial arterial diseases/ or cerebral arterial diseases/ or infarction, anterior cerebral artery/ or infarction, middle cerebral artery/ or infarction, posterior cerebral artery/ or exp intracranial embolism/) and thrombosis/) or exp stroke/ or vertebral artery dissection/
31	(isch?emi$ adj6 (stroke$ or apoplex$ or cerebral vasc$ or cerebrovasc$ or cva or attack$)).tw.
32	((brain or cerebr$ or cerebell$ or vertebrobasil$ or hemispher$ or intracran$ or intracerebral or infratentorial or supratentorial or middle cerebr$ or mca$ or anterior circulation) adj5 (isch?emi$ or infarct$ or thrombo$ or emboli$ or occlus$ or hypoxi$)).tw.
33	30 or 31 or 32

ID	Search
34	exp coronary disease/
35	exp myocardial infarction/
36	exp angina pectoris/
37	coronary.tw.
38	angina.tw.
39	myocardial infarction.tw.
40	34 or 35 or 36 or 37 or 38 or 39
41	33 or 40
42	5 and 29 and 41

Embase search strategy

(1974 to July 2017)

ID	Search
1	thienopyridine derivative/
2	thienopyridine$.tw.
3	(clopidogrel or prasugrel or ticagrelor).tw.
4	(ADP adj3 receptor antagonist*).tw,ot.
5	Randomized Controlled Trial/
6	double-blind method/
7	single-blind method/
8	randomized controlled trial:.mp.
9	((singl$ or double$ or trebl$ or tripl)*adj*(*blind* or mask$)).mp.
10	controlled clinical trial/
11	Clinical Trial/
12	exp comparative study/
13	follow up/
14	prospective study/
15	random:.mp.
16	(random: adj5 (controlled or clinical)).mp.
17	11 or 12 or 13 or 14
18	15 or 16
19	17 and 18
20	5 or 6 or 7 or 8 or 9 or 10 or 19
21	exp animal/
22	nonhuman/
23	exp animal experiment/
24	21 or 22 or 23
25	exp human/
26	24 not 25
27	20 not 26
28	cerebrovascular disease/ or brain infarction/ or brain stem infarction/ or cerebellum infarction/ or exp brain ischemia/ or carotid artery disease/ or exp carotid artery obstruction/ or cerebral artery disease/ or exp cerebrovascular accident/ or exp occlusive cerebrovascular disease/ or stroke patient/
29	(isch?emi$ adj6 (stroke$ or apoplex$ or cerebral vasc$ or cerebrovasc$ or cva or attack$)).tw.
30	((brain or cerebr$ or cerebell$ or vertebrobasil$ or hemispher$ or intracran$ or intracerebral or infratentorial or supratentorial or middle cerebr$ or mca$ or anterior circulation) adj5 (isch?emi$ or infarct$ or thrombo$ or emboli$ or occlus$ or hypoxi$)).tw.
31	exp Coronary Artery Disease/

ID	Search
32	exp Coronary Artery Obstruction/
33	exp coronary artery/
34	exp heart infarction/
35	exp angina pectoris/
36	coronary.tw.
37	angina.tw.
38	myocardial infarction.tw.
39	28 or 29 or 30
40	31 or 32 or 33 or 34 or 35 or 36 or 37 or 38
41	39 or 40
42	1 or 2 or 3 or 4
43	27 and 41 and 42

CENTRAL search

ID	Search
#1	[mh Thienopyridines] or [mh “Purinergic P2Y Receptor Antagonists”]
#2	thienopyridine*:ti,ab,kw
#3	clopidogrel or prasugrel or ticagrelor:ti,ab,kw
#4	{or #1-#3}
#5	CORONARY DISEASE
#6	CORONARY CIRCULATION
#7	MYOCARDIAL REVASCULARIZATION
#8	CORONARY VESSELS
#9	coronary
#10	angina
#11	(myocardial next infarction)
#12	(heart next infarction)
#13	{or #5-#12}
#14	[mh “cerebrovascular disorders”] or [mh “basal ganglia cerebrovascular disease”] or [mh “brain ischemia”] or [mh “brain infarction”] or [mh “hypoxia-ischemia, brain”] or [mh “carotid artery diseases”] or [mh “carotid artery thrombosis”] or [mh “carotid artery, internal, dissection”] or [mh “intracranial arterial diseases”] or [mh “cerebral arterial diseases”] or [mh “infarction, anterior cerebral artery”] or [mh “infarction, middle cerebral artery”] or [mh “infarction, posterior cerebral artery”] or [mh “intracranial embolism and thrombosis”] or [mh stroke] or [mh “vertebral artery dissection”]
#15	isch_mi_ near/6 (stroke* or apoplex* or cerebral next vasc* or cerebrovasc* or cva or attack*):ti,ab,kw (Word variations have been searched)
#16	(brain or cerebr* or cerebell* or vertebrobasil* or hemispher* or intracran* or intracerebral or infratentorial or supratentorial or middle next cerebr* or mca _or “anterior circulation”) near/5 (isch_mi* or infarct* or thrombo* or emboli _or occlus_ or hypoxi*):ti,ab,kw
#17	#14 or #15 or #16
#18	#13 or #17
#19	#4 and #18

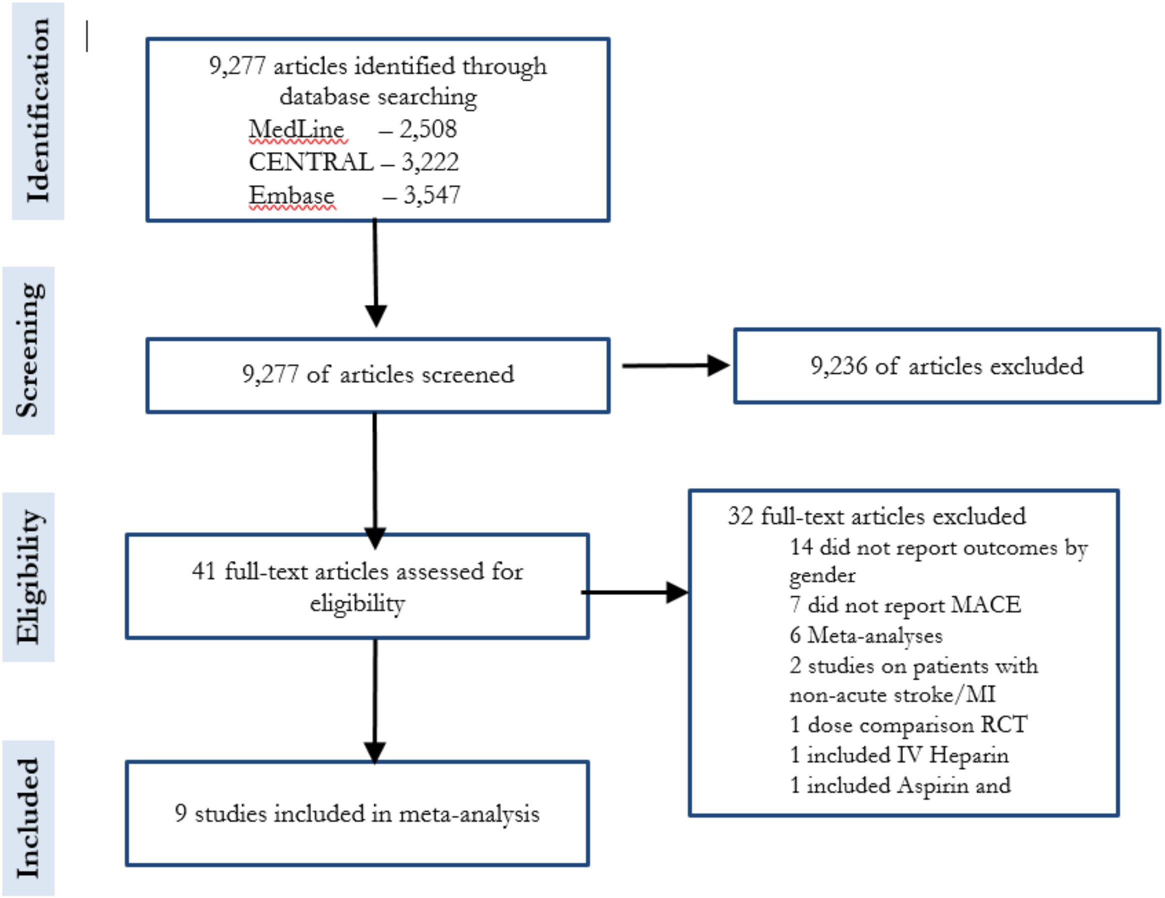
Identification
Screening
Eligibility
Included
9,277 articles identified through database searching
MedLine – 2,508
CENTRAL – 3,222
Embase – 3,547
9,277 of articles screened
9,236 of articles excluded
41 full-text articles assessed for eligibility
32 full-text articles excluded
14 did not report outcomes by gender
7 did not report MACE
6 Meta-analyses
2 studies on patients with non-acute stroke/MI
1 dose comparison RCT
1 included IV Heparin
1 included Aspirin and
9 studies included in meta-analysis

Figure 1:

Consort diagram

World Health Organisation ICD-10 codes used to identify bleeding deaths

Deaths from bleeding were defined if these codes appeared in any position.

K22.6, K62.5, K92.0, K92.1, K92.2, N83.7, N93.8, N93.9, O03.1, O03.6, O04.1, O04.6, O05.1, O05.6, O06.1, O07.1, O07.6, O08.1, O20.8, O20.9, O46, O71.7, O90.2, R04.2, T81.0, K25.0, K25.4, K26.0, K26.4, K27.0, K27.4, K28.0, K28.4, K29.0, O67, O72.0, O72.1, O72.2, P26.1, R04.1, R04.8, R04.9, I85.0, K25.2, K25.6, K26.2, K27.2, K27.6, K28.2, K28.6, H35.6, H43.1, H45.0, I60, I62, 169.0, 169.2, S06.4, S06.5, S06.6, I23.0, I31.2.

Trial participant baseline characteristics

	CURE	COMMIT	CLARITY-TIMI 28	TRITON-TIMI 38
Demographics				
Female Sex	38.5	27.8	19.7	26
Age (mean)	64.2	61.3	57.5	61
Age (SD)	11.3	11.85	10.3	
Older		>=70: 26		>=75: 13
Medical History				
Smoker	60.7		50.2	38
Diabetes Mellitus	22.6		16.5	23
Hypertension	58.8	43.3	43.4	64
Hyperlipidaemia			32.6	56
Previous MI	32.2	8.3	9.1	18
Previous PCI			4.8	
Previous CABG				7.5
Previous PCI or CABG	17.9			
Previous Stroke				
Previous TIA				
Diagnosis				
Unstable Angina	74.9			
NSTEMI	25.1	6.9		
Unstable Angina or NSTEMI				74
STEMI		93.1	100.0	26
TIA				
Ischaemic Stroke				

	PLATO	CHANCE	TRILOGY ACS	SPS3
Demographics				
Female Sex	28.4	33.8	35.9	37.0
Age (mean)	62	62.5	62	63.0
Age (SD)				
Older	>=75: 15.5	>=71.5: 25	>=75: 22	
Medical History				
Smoker	35.9	43	23.4	20.5
Diabetes Mellitus	25	21.1	38.9	36.5

	PLATO	CHANCE	TRILOGY ACS	SPS3
Hypertension	65.4	65.7	80.4	75.0
Hyperlipidaemia	46.7	11.1	59	
Previous MI	20.5	1.9	44.1	
Previous PCI	13.4		28.1	
Previous CABG	5.9		15.4	
Previous PCI or CABG				
Previous Stroke		20		15.0
Previous TIA		3.4		
Diagnosis				
Unstable Angina	16.7		32.5	
NSTEMI	42.7		67.5	
Unstable Angina or NSTEMI				
STEMI	37.7			
TIA		27.9		3.0
Ischaemic Stroke		72.1		97.0

Presents percentages for categorical variables and means for continuous variables.

Trial effect estimates for acute coronary syndrome patients with and without unstable angina

Relative treatment effect estimates for main treatment effects, stratified by diagnosis, as reported in original trial publications

Trial	Exclude UA	Include UA	Overall
CURE	Associated MI – 0.82	No Associated MI – 0.82	0.80 (0.72 to 0.90)
PLATO	NSTEMI – 0.83, STEMI – 0.84	UA – 0.96	0.84 (0.77 to 0.92)
TRITON-TIMI 38	STEMI – 0.81	UA or NSTEMI – 0.81	0.81 (0.73 to 0.90)
TRILOGY ACS	NSTEMI – 0.91	UA – 0.92	0.91 (0.79 to 1.05)

UA - unstable angina, STEMI - ST-elevation MI, NSTEMI - non-ST elevation MI The effect estimates are risk ratios for CURE and TRITON-TIMI 38, and hazard ratios for PLATO and TRILOGY-ACS.

Sex-treatment interaction rate ratio

Rationale for modelling approach

As described in the methods section. We fitted 3 models for the sex-treatment interaction rate ratio; an identical interaction for all trials within the drug-class, shared interactions between trials within the drug-class (using the assumption of exchangeability of covariate-treatment interactions within drug-class), and shared interactions between trials having stratified by indication/treatment.

Rather than simply conduct a stratified model, we opted to include models which allowed information sharing between trials from different drug-classes as a measure to deal with the well-described problem that occurs when estimating covariate-treatment interactions; low sample size and event numbers, which results in poor precision, low power and an increased risk of false positives (Brookes et al, 2004).

We therefore examined for evidence of sex-treatment interaction for P2Y12 inhibitor drugs not at the level of individual drugs, but rather at the level of the drug-class. Indeed, based on the putative mechanisms for a sex-treatment interaction, we would argue that a class-effect is more plausible than an agent-specific effect. For example, as we mention in the discussion, there may be differential thrombogenic tendencies and platelet-subendothelial interactions following antiplatelet therapy in women compared to men. If such a mechanism modifies the effectiveness of P2Y12-inhibitors, it is likely to be an effect common to the drug-class.

Such an effect would also likely be common to acute stroke as well as acute myocardial infarction. These diseases also share similarities in terms of mechanisms of disease (atherothrombotic disease), mechanism of treatment (inhibition of P2Y12-inhibition), outcome of interest (major adverse cardiovascular events), major adverse events (bleeding) and patient populations (acutely ill patients recently admitted to hospital with a major cardiovascular illness), which is why we decided, a priori, to also include stroke trials.

Moreover, for the best-fitting model, on which all subsequent analyses were based, we did not assume that the sex-treatment comparison was the same for all trials, but only that these were exchangeable, ie knowing the sex-treatment interaction for any trial within this drug-class tells us something about the likely size and magnitude of the sex-treatment interaction for each treatment comparison.

Overall, the effect of this information sharing was conservative, the sex-treatment interaction rate ratio for prasugrel/ticagrelor versus clopidogrel from the stratified model (RR 1.11) was shrunk towards the drug-class average in the shared model (RR 1.08). Importantly, the result was also robust to the choice of model.

Results for each model with model information criteria.

	Interaction rate ratio	Deviance	pD	DIC
Identical	1.09 (1.01-1.17)	273	18	291
Shared	1.08 (0.98-1.19)	259	27	286
Shared, stratified by comparison	1.11 (0.93-1.32)	261	29	289

In an additional model, we explored an approach which was intermediate, in terms of information sharing, between the shared and stratified models. In this more complex model trial was nested within treatment comparison (as with the stratified model), but a single shared prior was used across the three treatment comparison estimates. For simplicity, we did not present the results from this intermediate model as the estimate was similar to the more extreme shared and stratified models.

For other examples of Bayesian approaches to analysing trial data which employ similar techniques see; Berry and Berry, 2004 who describe sharing information within physiological systems to detect adverse events, Dixon and Simon, 1991 and Jones et al., 2011 who describe sharing information in sub-group analyses for individual trials and meta-analyses, and Owen, Tincello and Keith, 2015 who demonstrate how information can be shared within drug-classes to improve precision in network meta-analysis.

Estimated sex-treatment interaction for each comparison obtained from the stratified model.

	Estimate	Lower CI	Upper CI
Clopidogrel versus placebo, ACS	1.08	0.91	1.28
Clopidogrel versus placebo, stroke	1.02	0.81	1.30
Prasugrel/Ticagrelor versus placebo, ACS	1.11	0.93	1.32

ACS - acute coronary syndrome, CI - 95% credible interval

Absolute risk reduction by cause for all analyses

The following series of plots show estimated absolute risk reduction at one-year for death from cardiovascular, bleeding and competing (non-cardiovascular, non-bleeding) causes, as well as all-cause mortality. Each small multiple represents the results from a model with different treatment effects from the main analysis and each of the sensitivity analyses. The left-hand column shows the results from the sex-treatment interaction for MACE estimated from the data. The right-hand column shows the results from an assumed null interaction. The rows show the estimated treatment effect for a range of different sex-treatment interactions for bleeding.

Cardiovascular death

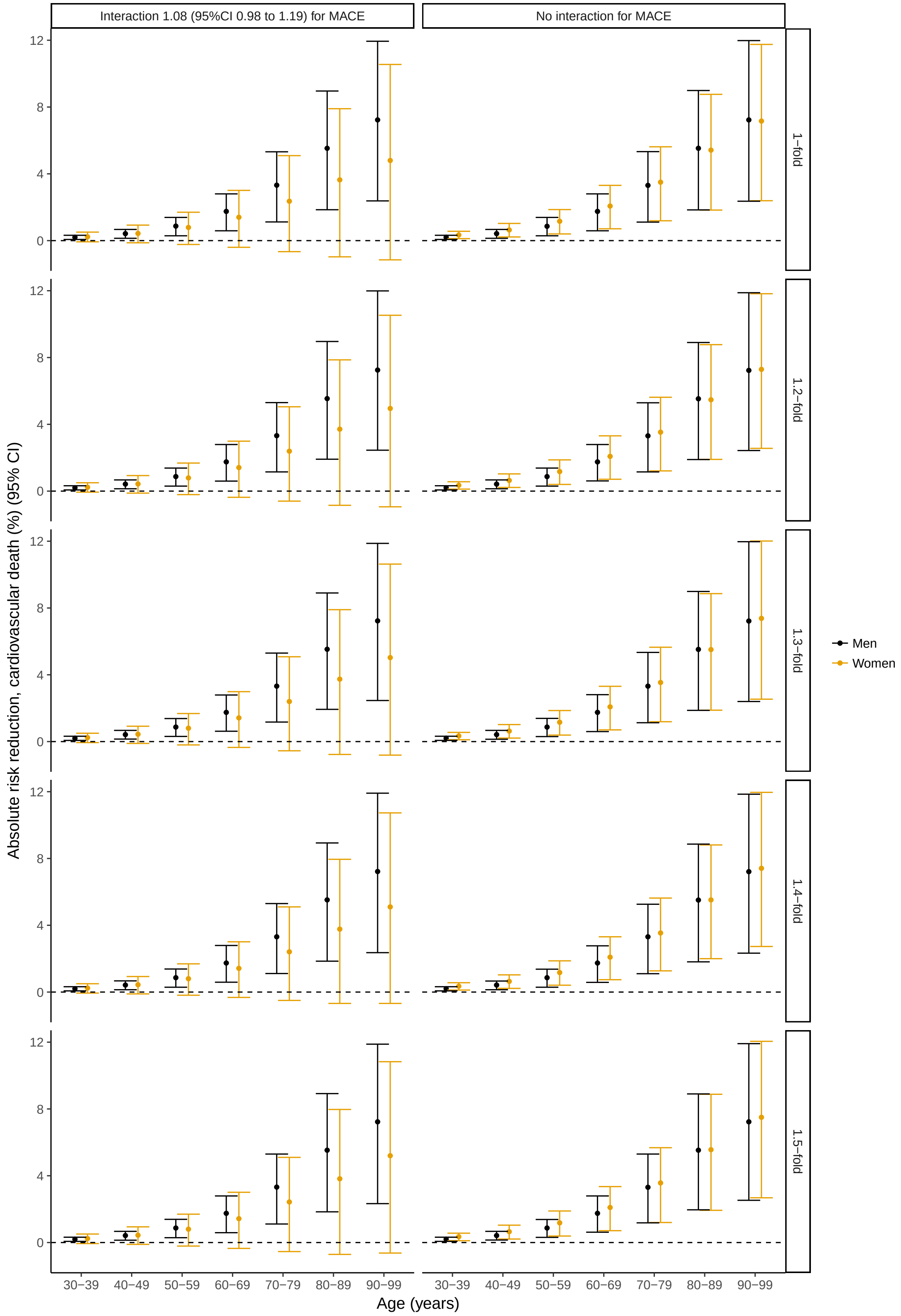

Bleeding deaths

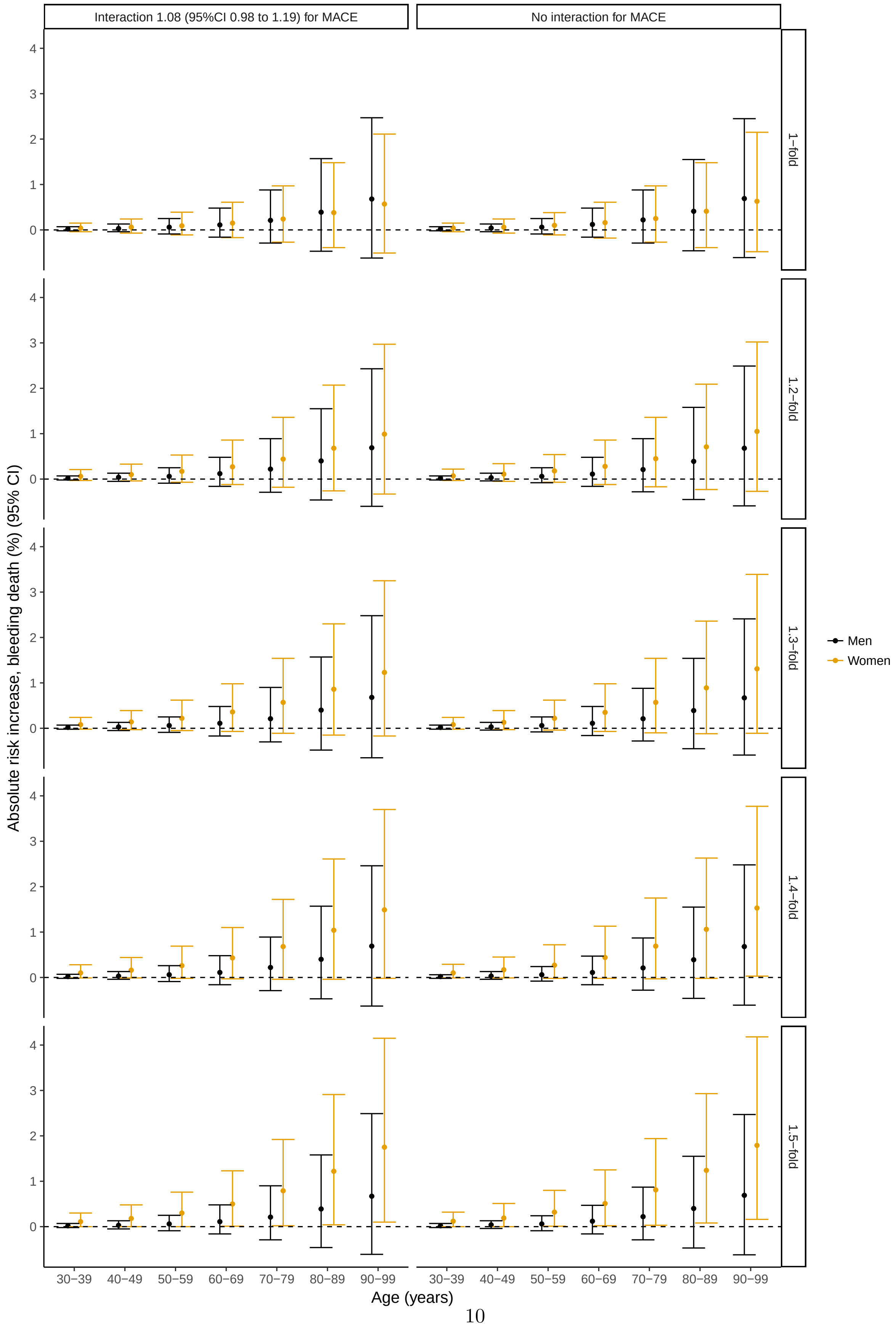

Non-cardiovascular, non-bleeding deaths

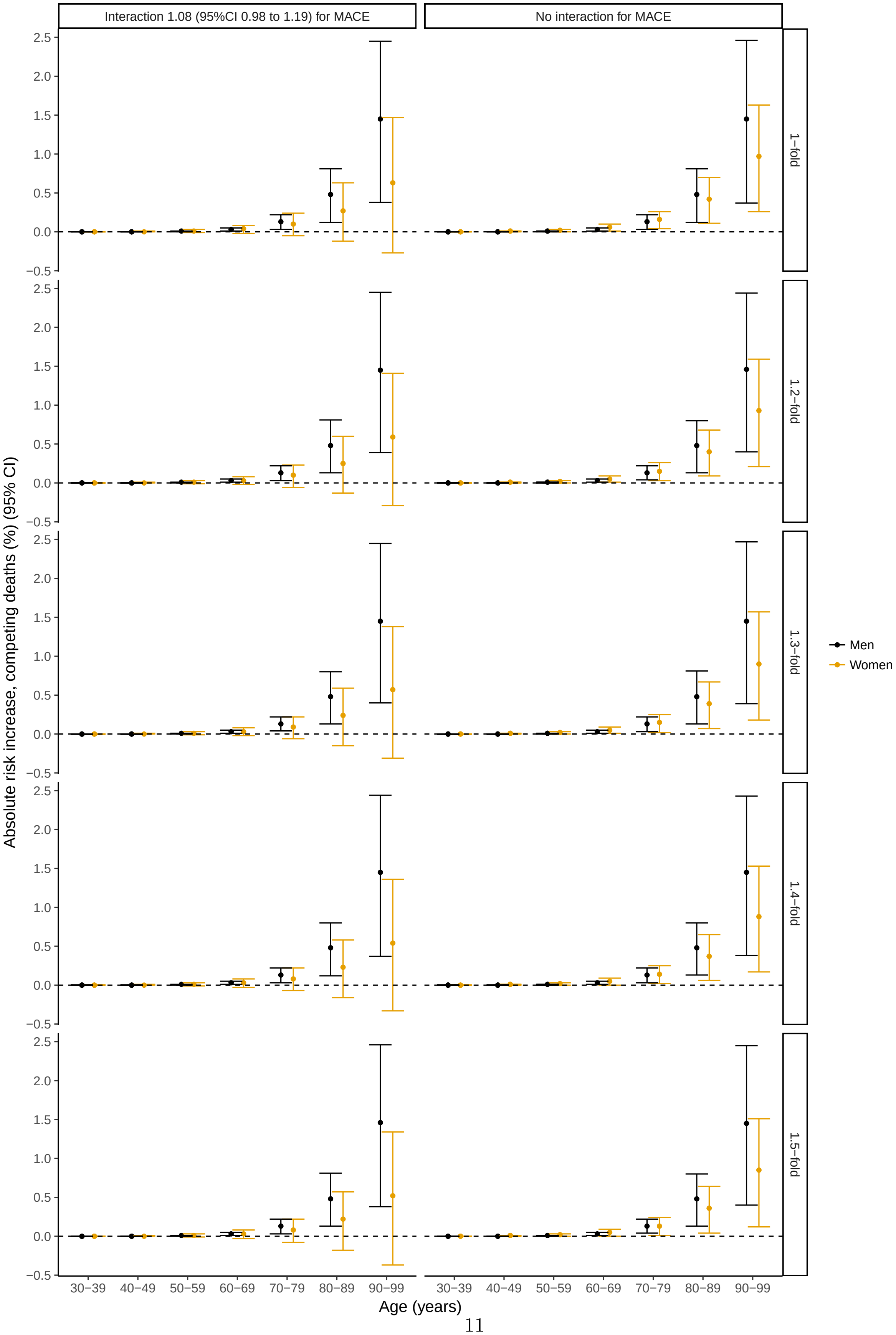

All-cause deaths

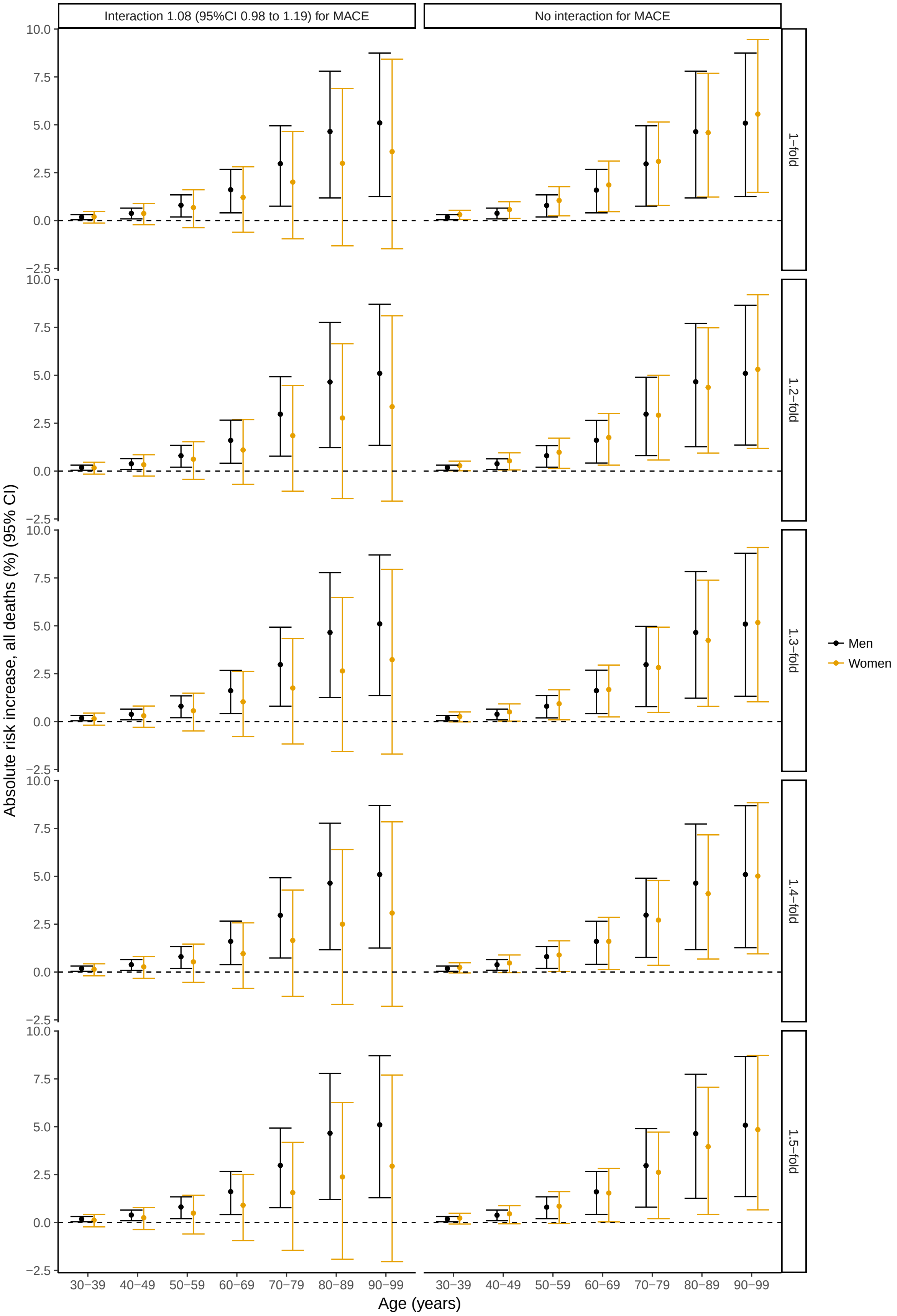

Re-analysis of data from recently published paper by Lau et al.

We re-analysed data provided in the Lau et al paper, as well as a combination of data obtained in our systematic review as well as the Lau et al paper using the “shared” model as described in the main manuscript.

	Trials	Sex-treatment interaction rate ratio
Main analysis	9	1.08 (0.98-1.19)
Lau et al	6	1.00 (0.86-1.14)
Both systematic reviews	12	1.05 (0.96-1.14)

Detailed model description

Relative treatment effects

The following equations define the shared model. In order to reduce the number of subscripts, and for compatibility with the structure of the data and comparability with the BUGS code, the likelihood and link functions are given separately for men and women allocated to the treatment and control arms. rmc, rwc, rmt and rwt represent the event numbers in men and women in the control groups and men and women in the treatment groups respectively. The number of participants in these groups are represented by nmc, nwc, nmt and nwt respectively. z indexes the trial. Consistent with the BUGS language, the following normal distributions are parameterised in terms of the mean and precision.

Likelihood and link function

Men controls

$$rmc_z \sim Bin(pmc_z, nmc_z)$$

$$cloglog(pmc_z) = log(time_z) + \mu_z$$

Women controls

$$rwc_z \sim Bin(pwc_z, nwc_z)$$

$$cloglog(pwc_z) = log(time_z) + mu_z + wu_z$$

Men treatment

$$rmt_z \sim Bin(pmt_z, nmt_z)$$

$$cloglog(pmt_z) = log(time_z) + mu_z + d_z$$

Women treatment

$$rwt_z \sim Bin(pwt_z, nwt_z)$$

$$cloglog(pwt_z) = log(time_z) + mu_z + wu_z + d_z + wd_z$$

Priors

Independent priors at the level of each trial were used for the intercept, female-sex and treatment effect in men. A shared prior was used for the sex-treatment interaction.

Intercept (ie baseline for each trial)

$$mu_z \sim N(0, 0.0001)$$

Effect of female sex

$$wu_z \sim N(0, 0.0001)$$

Treatment effect in men

$$d_z \sim N(0, 0.0001)$$

Shared across trials

Sex-treatment interaction

$$wd_z \sim N(wd\_delta, wd\_tau)$$

Meta-analysis level priors

Sex-treatment interaction across trials

$$wd\_delta \sim N(0, 0.0001)$$

Between-trial variation in sex-treatment interaction (precision)

$$wd\_tau = 1/wd\_var$$

Between-trial variation in sex-treatment interaction (variance)

$$wd\_var \sim lognormal(-3.23, 1/1.88^2)$$

A weakly informative prior was used for the between-trial variation in sex-treatment interaction (wd_var) as variance parameters are often difficult to fit and this parameter was not the main focus of the analysis. The $lognormal(-3.23, 1/1.88^2)$ prior was chosen from among the set of off-the-shelf priors from Turner et al 2012. Turner et al identified a set of priors for modelling heterogeneity in main treatment effects rather than interactions, and on the log-odds scale rather than the rate ratio scale. Nonetheless, we used this prior as it was felt to be sufficiently wide.

The likelihood and link functions were the same for the "identical" sex-treatment effect model. However, the sex-treatment interaction parameter wd was identical for all trials and so was not indexed and had a single prior ($N(0, 0.001)$).

The model stratified by the three treatment comparisons (clopidogrel versus placebo in stroke, clopidogrel versus placebo in ACS and ticagrelor or prasugrel versus clopidogrel in ACS) was also similar to this model. However,an additional index for treatment comparison was added to allow the sex-treatment interaction to vary between comparisons $wd\_delta_c \sim N(0, 0.0001)$. The between trial variance was assumed to be identical for all treatment comparisons.

Summarising treatment effects

Using samples from the posterior distributions from these models, treatment effects for men were added to the sex-treatment interaction (both on the log-RR scale) to estimate the sex-specific log-RR for women. The resultant sex-specific log-RRs for women and men were summarised using non-linear least squares in R, assuming a t-distribution.

Baseline risk model

We modelled cause-specific deaths from bleeding, cardiovascular disease and non-bleeding non-cardiovascular causes using a hierarchical generalized linear regression model with a multinomial likelihood and log link, as these outcomes are mutually exclusive.

In this model the i subscript refers to each row of the data, which is provided below the model. Each j subscript refers to each of the three outcomes.

Likelihood $y_{i,j} \sim Multinomial(p_{i,j}, N_i)$ with $\sum p_{i,j} = 1$

Linear predictor and link function

$$log(p_{i,j}) = \alpha0_j + \alpha1_j * age_i + \alpha2_j * gender_i$$

```
##      age gender    n bleed   cv other
## 1  30-39   Male  507     0    8     2
## 2  40-49   Male 2928     6   77    22
## 3  50-59   Male 5513    18  228    69
## 4  60-69   Male 6688    32  563   220
## 5  70-79   Male 6837    67 1299   557
## 6  80-89   Male 4653    90 1600   566
## 7  90-99   Male  692    15  342   110
## 8  30-39 Female  147     0    8     4
## 9  40-49 Female  793     0   22    16
## 10 50-59 Female 1681    13   84    40
## 11 60-69 Female 3109    13  312   161
## 12 70-79 Female 5066    48  990   437
## 13 80-89 Female 5518    91 1933   597
## 14 90-99 Female 1528    28  683   228
```

Application of relative treatment effects to baseline risk model to estimate absolute treatment effects.

Within JAGS we combined the estimates from the baseline model and the relative treatment effect models for the bleeding and cardiovascular outcomes to estimate the absolute risk reduction for cause-specific mortality from bleeding and from cardiovascular death as well as from all-cause mortality. For men and women, this was done through the following model.

$$log(pnew_{i,j}) = \alpha0_j + \alpha1_j * age_i + \alpha2_j * gender_i + men\_logrr_j + women\_logrr_j * gender_i$$

In this model the i subscript refers to each row of the data, as shown above. Also as before the j subscript refers to each of the three outcomes. men_logrr_j and $women\_logrr_j$ refer to the log-rate ratios for the effect of treatment in men and women respectively. From this model the absolute risk reduction for deaths from bleeding, cardiovascular and non-bleeding non-cardiovascular causes as well as for all causes were derived as follows.

$$arr_j = \frac{1}{\sum n_i} * \sum_i (n_i * (p_{i,j} - pnew_{i,j}))$$

$$arr = \sum_j arr_j$$

Model assumptions

The following assumptions were required for this analysis:-

- Sex-treatment interactions are exchangeable between trials within the same drug-class
- Sex-treatment interactions are homogeneous, on the relative scale, across follow-up time
- Sex-specific relative treatment effects are transportable from the clinical trial populations to the target Scottish population

- For the age-specific analysis that non-collapsability does not importantly affect the age-specific rate ratios
- That the rate ratio for non-cardiovascular, non-bleeding events is null.
- That the rate ratios for TIMI bleeding and MACE are the same as the rate ratios for bleeding death and cardiovascular death respectively. This involves the following assumptions:-
  - that bleeding deaths within one year cannot occur without TIMI major bleeding within one-year (with a similar assumption for MACE and cardiovascular deaths). This assumption is actually true by definition as TIMI major bleeding and MACE both include death from bleeding and cardiovascular death respectively as part of their outcome definitions
  - that the proportion of TIMI bleeding events which are bleeding deaths is not altered by treatment (ie that treatment may modify the proportion of patients who experience bleeding within one-year, but not the spectrum of bleeding severity) and that the proportion of MACE events which are cardiovascular deaths are similarly not altered by the treatment (ie that conditional on conditional on having a bleeding (or MACE event), the risk of a bleeding (or cardiovascular) death is not related to treatment)

Model code

Identical sex-treatment interaction model

```
##  [1] model{
##  [2]   for (z in 1:n_trials){
##  [3]     # likelihood
##  [4]     rmc[z] ~ dbin(pmc[z],nmc[z]) # men controls
##  [5]     rmt[z] ~ dbin(pmt[z],nmt[z]) # men treated
##  [6]     rwc[z] ~ dbin(pwc[z],nwc[z]) # women controls
##  [7]     rwt[z] ~ dbin(pwt[z],nwt[z]) # women treated
##  [8]
##  [9]     # link and linear predictor
## [10]     cloglog(pmc[z]) <- log(time[z]) +  mu[z]                      # baseline in men
## [11]     cloglog(pwc[z]) <- log(time[z]) +  mu[z] + wu[z]              # baseline in women
## [12]     cloglog(pmt[z]) <- log(time[z]) +  mu[z] +         d         # treatment in men
## [13]     cloglog(pwt[z]) <- log(time[z]) +  mu[z] + wu[z] + d + wd    # treatment in women
## [14]
## [15]     # Trial specific priors
## [16]     mu[z] ~ dnorm (0, 0.0001)
## [17]     wu[z] ~ dnorm (0, 0.0001)
## [18]
## [19]     } #end of trials loop
## [20]     # Shared priors (fixed effects)
## [21]     d  ~ dnorm (0, 0.0001)
## [22]     wd ~ dnorm (0, 0.0001)
## [23] }# end of model
```

Shared sex-treatment interaction model

```
##  [1] model{
##  [2] for (z in 1:n_trials){
##  [3]    # likelihood
##  [4]    rmc[z] ~ dbin(pmc[z],nmc[z]) # men controls
##  [5]    rmt[z] ~ dbin(pmt[z],nmt[z]) # men treated
```

```
##  [6]    rwc[z] ~ dbin(pwc[z],nwc[z]) # women controls
##  [7]    rwt[z] ~ dbin(pwt[z],nwt[z]) # women treated
##  [8]
##  [9]    # link and linear predictor
## [10]    cloglog(pmc[z]) <- log(time[z]) +  mu[z]                        # baseline in men
## [11]    cloglog(pwc[z]) <- log(time[z]) +  mu[z] + wu[z]                # baseline in women
## [12]    cloglog(pmt[z]) <- log(time[z]) +  mu[z] +        d[z]          # treatment in men
## [13]    cloglog(pwt[z]) <- log(time[z]) +  mu[z] + wu[z] + d[z] + wd[z] # treatment in women
## [14]
## [15]    # Trial specific priors
## [16]    mu[z] ~ dnorm (0, 0.0001)
## [17]    wu[z] ~ dnorm (0, 0.0001)
## [18]    d[z]  ~ dnorm (0, 0.0001)
## [19]    # Shared priors (random effects)
## [20]    wd[z] ~ dnorm(wd_delta, wd_tau)
## [21] } #end of trials
## [22]    # Meta-analysis level priors
## [23]
## [24]    wd_delta ~ dnorm(0, 0.0001)
## [25]    wd_tau <-  1/wd_var
## [26]    wd_var ~ dlnorm(-3.23, 1/1.88^2)
## [27]    wd_sd <- wd_var^0.5
## [28]
## [29]    #Residual deviance for all trials
## [30]   # totresdev <- sum(resdev[]) #Total Residual Deviance
## [31] }# end of model
```

Shared sex-treatment interaction model, stratified by treatment comparison

```
##  [1] model{
##  [2]   for(ci in 1:Ncompar_indic) {
##  [3]     for(z in Scompar_indic[ci]:(Scompar_indic[ci+1]-1)){
##  [4]       # likelihood
##  [5]       rmc[z] ~ dbin(pmc[z],nmc[z]) # men controls
##  [6]       rmt[z] ~ dbin(pmt[z],nmt[z]) # men treated
##  [7]       rwc[z] ~ dbin(pwc[z],nwc[z]) # women controls
##  [8]       rwt[z] ~ dbin(pwt[z],nwt[z]) # women treated
##  [9]       # link and linear predictor
## [10]       cloglog(pmc[z]) <- log(time[z]) +  mu[z]                         # baseline in men
## [11]       cloglog(pwc[z]) <- log(time[z]) +  mu[z] + wu[z]                 # baseline in women
## [12]       cloglog(pmt[z]) <- log(time[z]) +  mu[z] +        md[z]          # treatment in men
## [13]       cloglog(pwt[z]) <- log(time[z]) +  mu[z] + wu[z] + md[z] + wd[z] # treatment in women
## [14]       # Trial specific priors
## [15]       mu[z] ~ dnorm (0, 0.0001)
## [16]       wu[z] ~ dnorm (0, 0.0001)
## [17]       md[z]  ~ dnorm (0, 0.0001)
## [18]       wd[z] ~ dnorm(wd_delta[ci],prec1)
## [19]     }#trials
## [20]     wd_delta[ci] ~ dnorm(0, 0.0001)
## [21]   }#comparison and indication
## [22]   var1 ~  dlnorm(-3.23, 1/1.88^2)
## [23]   prec1 <- 1 / var1
## [24]   sd1 <- var1^0.5
```

```
## [25] } # model
```

References

Berry, S. M. and Berry, D. A. (2004) 'Accounting for Multiplicities in Assessing Drug Safety: A Three-Level Hierarchical Mixture Model', Biometrics, 60(2), pp. 418–426. doi: 10.1111/j.0006-341X.2004.00186.x.

Brookes ST, Whitely E, Egger M, et al. Subgroup analyses in randomized trials: risks of subgroup-specific analyses; power and sample size for the interaction test. J Clin Epidemiol 2004;57:229–36. doi: 10.1016/j.jclinepi.2003.08.009

Dixon, D. O. and Simon, R. (1991) 'Bayesian Subset Analysis', Biometrics, 47(3), pp. 871–881. doi: 10.2307/2532645.

Jones HE, Ohlssen DI, Neuenschwander B, et al.Bayesian models for subgroup analysis in clinical trials. Clin Trials 2011;8:129–43. doi:10.1177/1740774510396933

Owen RK, Tincello DG, Keith RA. Network Meta-Analysis: Development of a Three-Level Hierarchical Modeling Approach Incorporating Dose-Related Constraints. Value Health 2015;18:116–26. doi:10.1016/j.jval.2014.10.006.

Turner, Rebecca M, Jonathan Davey, Mike J Clarke, Simon G Thompson, and Julian PT Higgins. "Predicting the Extent of Heterogeneity in Meta-Analysis, Using Empirical Data from the Cochrane Database of Systematic Reviews." International Journal of Epidemiology 41, no. 3 (June 2012): 818–27. doi:10.1093/ije/dys041.
